# Refractive outcome and tomographic changes after Descemet membrane endothelial keratoplasty in pseudophakic eyes with Fuchs’ endothelial dystrophy

**DOI:** 10.1007/s10792-021-01850-w

**Published:** 2021-06-29

**Authors:** Bishr Agha, Nura Ahmad, Daniel G. Dawson, Thomas Kohnen, Ingo Schmack

**Affiliations:** 1 Department of Ophthalmology, Goethe-University, Theodor-Stern-Kai 7, 60590 Frankfurt am Main, Germany; 2 Department of Ophthalmology, University of Florida, Gainesville, USA

**Keywords:** DMEK, Refractive changes, Corneal tomography, Pseudophakia

## Abstract

**Purpose:**

To analyze refractive and topographic changes secondary to Descemet membrane endothelial keratoplasty (DMEK) in pseudophakic eyes with Fuchs’ endothelial dystrophy (FED).

**Methods:**

Eighty-seven pseudophakic eyes of 74 patients who underwent subsequent DMEK surgery for corneal endothelial decompensation and associated visual impairment were included. Median post-operative follow-up time was 12 months (range: 3–26 months). Main outcome measures were pre- and post-operative manifest refraction, anterior and posterior corneal astigmatism, simulated keratometry (CA_SimK_) and *Q* value obtained by Scheimpflug imaging. Secondary outcome measures included corrected distance visual acuity (CDVA), central corneal densitometry, central corneal thickness, corneal volume (CV), anterior chamber volume (ACV) and anterior chamber depth (ACD).

**Results:**

After DMEK surgery, mean pre-operative spherical equivalent (± SD) changed from + 0.04 ± 1.73 D to + 0.37 ± 1.30 D post-operatively (*p* = 0.06). CDVA, proportion of emmetropic eyes, ACV and ACD increased significantly during follow-up. There was also a significant decrease in posterior corneal astigmatism, central corneal densitometry, central corneal thickness and corneal volume over time (*p* = 0.001). Only anterior corneal astigmatism and simulated keratometry (CA_SimK_) remained fairly stable after DMEK.

**Conclusion:**

Despite tendencies toward a hyperopic shift, changes in SE were not significant and refraction remained overall stable in pseudophakic patients undergoing DMEK for FED. Analysis of corneal parameters by Scheimpflug imaging mainly revealed changes in posterior corneal astigmatism pointing out the relevance of posterior corneal profile changes during edema resolution after DMEK.

## Introduction

Currently, corneal endothelial decompensation secondary to Fuchs’ endothelial dystrophy (FED) or pseudophakic bullous keratopathy (BKP) can be treated successfully by endothelial keratoplasty (i.e., Descemet membrane endothelial keratoplasty, DMEK) [1]. DMEK, which is focused on the selective replacement of a patient’s Descemet’s membrane and the damaged corneal endothelium, has some advantages compared to penetrating keratoplasty (PKP), like faster visual rehabilitation, superior visual outcome and lower rejection rate [2–5]. Lower rates of postoperative glaucoma and easier control of intraocular pressure are further advantages compared to penetrating keratoplasty [6]. Usually a high percentage of patients seeking DMEK surgery demonstrate cataractous changes of the crystalline lens as well or are already pseudophakic at the time of surgery. Accurate prediction of the refractive outcome in patients undergoing DMEK surgery is still an unsolved problem, especially patients with combined DMEK surgery and cataract surgery (triple procedure), who often times experience an unpredictable post-operative hyperopic shift [7–10]. Currently, it is thought that changes to the posterior corneal profile due to resolution of the stroma edema may have the highest impact on the refraction outcome [11]. To better understand these changes, assessment of the posterior corneal profile, best represented by the *Q* value, is assumed to be the most useful in estimating and understanding the postoperative hyperopic shift more precisely. Nevertheless, changes to additional topographic parameters that may also affect the postoperative refraction still need to be defined [12]. Since a high portion of DMEK patients already had previous cataract surgery, it is of interest to better understand if and how the final refractive outcome might be affected by postoperative corneal changes in these patients first since triple procedure patients have more complicated variables to consider and evaluate.

The purpose of our study was to evaluate refractive and corneal changes in pseudophakic patients undergoing DMEK surgery for FED. Different topographic parameters were analyzed regarding their potential contribution on post-operative refractive result in DMEK patients.

## Patients and methods

### Inclusion criteria

Medical records of 185 consecutive pseudophakic patients, who underwent DMEK surgery for FED between February 2015 and July 2018 at our department, were reviewed retrospectively. The study protocol was approved by the institutional review board of the Goethe-University and was in accordance with the tenets of the Declaration of Helsinki.

All DMEK surgeries were performed by two experienced surgeons (TK and IS) following the protocol by Melles [2]. Inclusion criteria were a minimum follow-up time of 3 months after DMEK, stable postoperative refraction, existence of pre- and post-operative Scheimpflug images and absence of previous corneal surgery.

### Pre- and post-DMEK assessment

Demographic data, CDVA, target refraction after previous cataract surgery and ocular comorbidities were extracted from medical records. Central corneal thickness (CCT), corneal astigmatism, average keratometric readings of the anterior (KmF) and posterior surface (KmB), corneal volume, posterior *Q*-value, corneal densitometry and anterior chamber depth and volume were evaluated using a rotating Scheimpflug camera system (Pentacam AXL, Oculus GmbH, Wetzlar, Germany).

Corneal astigmatism assessment included magnitude and axis orientation at the front and back surface as well as simulated keratometry (CA_SimK_), a value for estimation of total corneal astigmatism determined by anterior corneal measurement only.

For analysis of the distribution of axis orientations of the corneal astigmatism, the following definitions described by Kamiya and co-workers were used:

- Anterior astigmatism: with-the-rule (WTR: steep meridian within 60–120°), against-the-rule (ATR: steep meridian within 0–30° or 150–180°) and oblique (steep meridian within 30–60° or 120–150°).
- Posterior astigmatism: WTR (steep meridian within 0–30° or 150–180°), ATR (steep meridian within 60–120°), the remaining astigmatism was classified as oblique astigmatism [13].

Posterior *Q* value, which describes the eccentricity of the posterior surface profile, can be positive (oblate cornea), zero (spheric cornea) or negative (prolate cornea). In the presence of stromal edema, the cornea tends to become more oblate (i.e., flatter centrally than peripherally, positive *Q* value).

Corneal densitometry, a parameter of corneal transparency, was assessed by corneal backscattered light measurements (grayscale units—GSU) of the entire cornea within three concentric corneal annular zones (0–2 mm zone, 2–6 mm zone, and 6–10 mm zone). Values ranged from 0 (completely transparent) to 100 (completely opaque).

### Statistical analysis

Statistical analysis was performed using Excel for Mac (version 15.37, Microsoft, Inc., Redmond, WA, USA) and SPSS version 25 (IBM Corp., Armonk, NY, USA). Normal distribution testing was done by using the Kolmogorov–Smirnov test. For normally distributed data, a Student´s t-test for paired values was performed. A two-sided Wilcoxon signed-rank test was used for a non-normal distribution. A *p*-value < 0.05 was considered statistically significant.

## Results

Inclusion criteria were met by 87 eyes of 74 patients (36 males and 38 females). The mean age (± SD) at the time of DMEK surgery was 71.7 ± 8.2 years (range: 47–91 years). Median follow-up time was 12 months (range: 3–26 months). The median time interval between previous cataract surgery and DMEK was 12 months (range: 2–112 months). Changes in postoperative CDVA (logMAR), refraction (spherical equivalent—SE), corneal topography, densitometry and anterior chamber are summarized in Table 1. The rebubbling rate was 17% (*n* = 15 eyes). Eyes with severe extracorneal visual limitations (amblyopia, macular degeneration or advanced glaucoma) were excluded from the statistical analysis of CDVA (*n* = 7).

**Table 1:** Clinical and Scheimpflug parameters (mean ± standard deviation (SD), range) before (preop) and after DMEK (postop)

Parameters	Preoperative(mean ± SD) (range)	Postoperative(mean ± SD) (range)	*P*
CDVA (logMAR)	0.68 ± 0.66 (0.1–3.0)	0.30 ± 0.29 (0–1.6)	< 0.001
S (D)	0.64 ± 1.71 (− 4.00 to 5.75)	0.93 ± 1.45 (− 2.25 to 6.0)	0.12
Cyl (D)	− 1.36 ± 1.06 (− 5.5 to 0)	− 1.22 ± 1.08 (− 6.5 to 0)	0.48
SE (D)	0.04 ± 1.73 (− 4.75 to 5.75)	0.37 ± 1.30 (− 2.75 to 4.75)	0.06
CA_ant_ (D)	1.44 ± 1.19 (0.1–5.3)	1.44 ± 1.08 (0–5)	0.36
CA_post_ (D)	0.59 ± 0.56 (0–3)	0.39 ± 0.27 (0–1.8)	0.001
CA_SimK_ (D)	1.46 ± 1.2 (0.1–5.3)	1.45 ± 1.08 (0–5)	0.44
KmF (D)	43.4 ± 1.9 (39.6–48.8)	43.2 ± 1.7 (39.2–46.9)	0.96
KmB (D)	− 5.72 ± 0.83 (− 8.3 to − 2.6)	− 6.33 ± 0.31 (− 7.2 to − 5.6)	< 0.001
Q post	0.08 ± 0.57 (− 1.1 to 1.51)	− 0.20 ± 0.33 (− 1.01 to 0.67)	< 0.01
CV (mm^3^)	65.2 ± 10.5 (55.7 to 114)	60.3 ± 5.9 (50.5–89.6)	< 0.001
ACV (mm^3^)	170.5 ± 56.3 (29 to 490)	175.9 ± 36.1 (19–238)	< 0.01
ACD (mm)	3.47 ± 0.81 (1.41 to 4.96)	4.04 ± 0.55 (1.81–4.95)	< 0.001
CCT (µm)	668 ± 118 (526–1232)	523 ± 44 (419–641)	< 0.001
CD (zone)			
0–2 mm	28.96 ± 9.68 (14.8–62.8)	18.84 ± 4.71 (12.1–35.2)	< 0.001
2–6 mm	25.57 ± 9.19 (14.1–65.9)	19.15 ± 5.52 (12.5–40.1)	< 0.001
6–10 mm	32.05 ± 10.53 (14.2–56.1)	30.5 ± 9.65 (12.7–57.7)	0.27

*CDVA* corrected distance visual acuity, *S* sphere, *Cyl* cylinder, *SE* spherical equivalent, *D* diopters, *CA*_*ant*_ anterior corneal astigmatism, *CA*_*post*_ anterior corneal astigmatism, *CA*_*SimK*_ simulated keratometric astigmatism, *KmF* average keratometric readings of the anterior surface, *KmB* average keratometric readings of the posterior surface, *Q post* posterior *Q* value, *CV* corneal volume, *ACV* anterior chamber volume, *ACD* anterior chamber depth, *CCT* central corneal thickness, *CD* corneal densitometry (total layer)

After DMEK surgery, CDVA (logMAR) improved significantly from 0.68 ± 0.66 to 0.30 ± 0.29 at the end of observation period (*p* < 0.001).

**Fig. 1:**
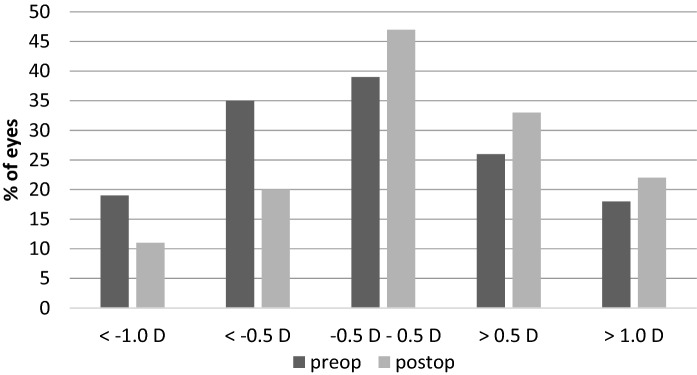
Distribution (%) of the spherical equivalent (SE) before (preop) and after (postop) DMEK. Preoperative refractive values were obtained from *n* = 72 eyes, postoperative values were obtained from *n* = 87 eyes, respectively

**Fig. 2:**
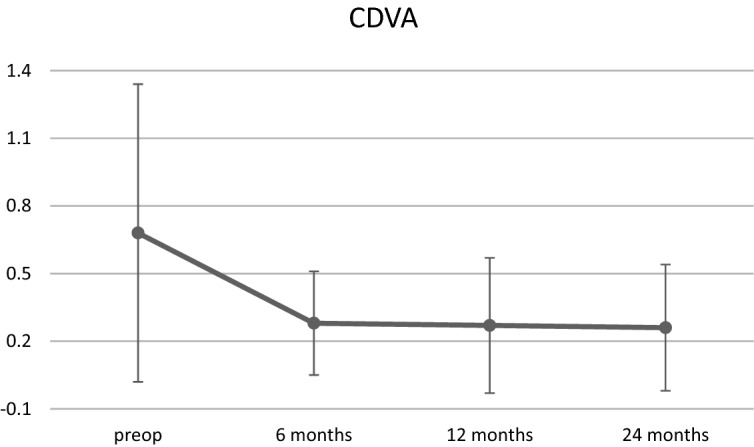
Changes in corrected distance visual acuity (CDVA) over time. Error bars depicting ± SD

**Fig. 3:**
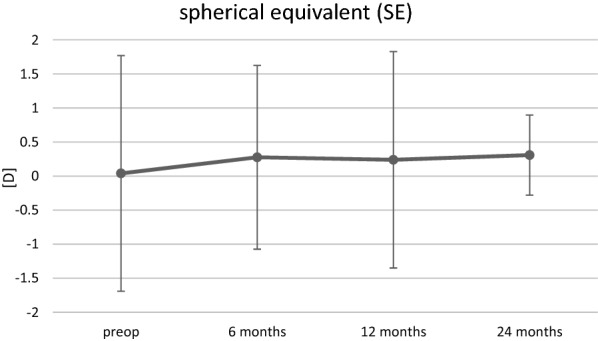
Changes in spherical equivalent (SE). Error bars depicting ± SD

Preoperatively, the majority of study eyes (39%) were emmetropic with a spherical equivalent (SE) ranging between  − 0.50 and + 0.50 diopters (D) as shown in Fig. 1. The percentage of myopic (<  − 0.50 D) and hyperopic (> 0.50 D) eyes was 35% and 26%, respectively. After DMEK surgery, mean SE (± SD) increased from + 0.04 ± 1.73 D to + 0.37 ± 1.30 D at the final follow-up visit. Although there was a slight hyperopic shift in all three subgroups, the total refractive changes were statistically not significant (p = 0.06). Overall, the percentage of myopic eyes (SE < −0.5 D) decreased from 35 to 20%. In contrast, the percentage of emmetropic and hyperopic eyes increased by 8% (39% to 47%) and 7% (26% to 33%), respectively (Fig. 1). CDVA and SE demonstrated changes up to 6 months after DMEK surgery and remained relatively stable afterwards (Figs. 2 and 3).

**Fig. 4:**
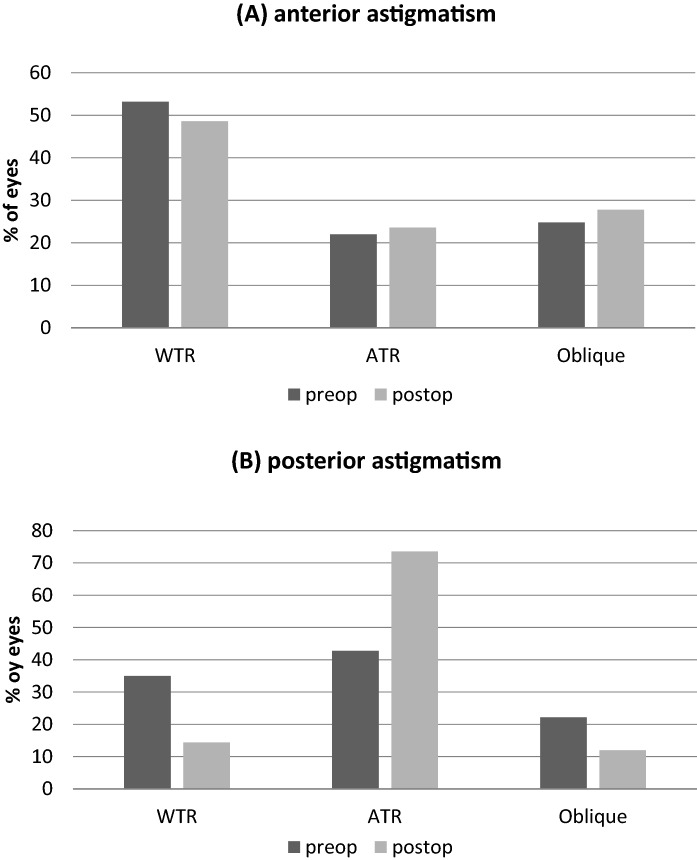
Axis orientations (%) of anterior (**a**), and posterior (**b**) corneal astigmatism before (preop) and after DMEK (postop). ATR = against-the-rule astigmatism; Oblique = oblique astigmatism; WTR = with-the-rule astigmatism

Topographic parameters, such as anterior corneal astigmatism and simulated keratometry (CA_SimK_) did not show any significant changes (Table 1). With-the-rule astigmatism (WTR) was present in the majority of eyes (53.2%). Postoperative changes were primarily limited to the posterior corneal surface only. For example, posterior corneal astigmatism decreased from + 0.59 ± 0.56 to + 0.39 ± 0.27 D (*p* = 0.001). In addition, the proportion of eyes demonstrating an against-the-rule (ATR) astigmatism increased from 42.8 to 73.6%, while the amount eyes with WTR and oblique astigmatism decreased after DMEK. Changes in the distribution of axis orientations of anterior and posterior corneal astigmatism are also shown in Fig. 4.

There was also a significant reduction in the corneal volume after DMEK (*p* < 0.001). Simultaneously, anterior chamber depth and volume increased significantly (Table 1). On the other hand, KmF remained fairly stable after surgery (*p* = 0.96). In contrast, KmB and CCT changed significantly (*p* < 0.001), with an increase (KmB) from −5.72 ± 0.83 to −6.33 ± 0.31 D (*p* < 0.001) and a decrease (CCT) from 667.9 ± 117.5 to 523.3 ± 43.6 µm (*p* < 0.001), respectively.

Corneal densitometry showed a significant reduction for the total layer in the 0–2 mm and 2–6 mm corneal annular zones (*p* < 0.001), while changes in the peripheral 6–10 mm zone were statistically not significant (*p* = 0.27).

## Discussion

In this study, we analyzed pseudophakic eyes for potential postoperative refractive and topographic changes secondary to DMEK surgery for FED. Currently, there are only few data available focusing on the refractive outcome in patients with previous cataract surgery. As optimization of refractive results is becoming more and more relevant for patients undergoing corneal surgery, additional data are helpful for a good surgical management[7]. Furthermore, only minor refractive changes after DMEK surgery in pseudophakic eyes can be an argument in favor for a sequential management (1st step: cataract extraction; 2nd step: DMEK surgery) over triple procedure, which has a reportedly high likelihood of a clinical relevant hyperopic shift [8–10, 16].

Overall, we also found a hyperopic shift in pseudophakic eyes after DMEK surgery; however, changes were only minor and statistically not significant. Our results were comparable with a study performed by van Dijk et al. which analyzed the refractive outcome of pseudophakic eyes after DMEK over a period of 2 years [14]. The authors were able to demonstrate that major changes were related to the anterior corneal curvature, showing slight changes over time. In contrast to the aforementioned study, the refractive astigmatism, anterior astigmatism and KmF remained almost stable in our study cohort.

In general, prediction of the post-operative refractive outcome is still an unresolved issue in patients with DMEK surgery for FED [1, 12]. A study investigating changes in the postoperative refraction after DMEK in phakic eyes (*n* = 52) found a mean hyperopic shift of + 0.74 D [15]. Another study, performed by Schoenberg and colleagues, was able to detect a refractive error of + 0.43 D following triple DMEK procedures in 108 eyes [16]. The authors concluded that a target refraction of − 0.75 to − 1.00 D might be helpful in reducing the proportion of eyes with postoperative hyperopia [16]. We only partially interpret the relatively high number of study eyes being not emmetropic after previous cataract surgery (53%) as a result of the narrow definition of emmetropia (± 0.5 D). Nevertheless, the percentage of eyes with postoperative emmetropia was still higher compared to a study investigating risk factors for hyperopia after DMEK surgery with combined cataract surgery (triple DMEK procedure) (47% vs. 38%) [12]. Taking into account the individual situations in which DMEK surgery might be performed (single DMEK in phakic eyes, single DMEK in pseudophakic eyes and simultaneous DMEK and cataract surgery), the aforementioned studies and our study data suggest that hyperopic changes seem to be least pronounced after DMEK surgery in pseudophakic eyes. This is presumably because inaccurate IOL power calculation due to resolving corneal edema after DMEK surgery has no longer to be considered. These findings might be an argument for choosing a step-wise procedural protocol in patients with cataract and endothelial dystrophy or limited endothelial pump function.

As already shown in previous studies, the anterior corneal profile remains fairly stable after DMEK surgery [17]. Therefore, reasons for postoperative hyperopic changes may be mainly related to modifications of the posterior corneal profile itself [18]. This assumption could be supported by our findings in pseudophakic FED showing no statistic significant changes in the anterior corneal astigmatism and KmF after DMEK surgery. However, we observed significant changes in regard to the posterior corneal astigmatism. Our findings are in accordance with a study performed by Yokogawa et al., which also showed a significant decrease in the magnitude of the posterior corneal astigmatism after DMEK surgery [19]. Along with a reduction of the magnitude of posterior corneal astigmatism, we also detected a shift in axis orientation to a higher proportion of eyes with ATR astigmatism, which is similar to results reported by Alnawaiseh et al. [20]. In a study with non-diseased, previously unoperated eyes, total corneal astigmatism could not be predicted adequately by anterior measurements only, especially in eyes that do not have with-the-rule astigmatism, pointing out the relevance of posterior corneal profile [21]. As is known in refractive surgery, posterior corneal astigmatism is a relevant parameter in IOL calculation to improve the postoperative refractive outcome [22, 23]. Unfortunately, reliable prediction of the post-operative astigmatism based on pre-operative measurements alone is not sufficiently possible in eyes with FED yet [24].

In a previous study investigating factors contributing to hyperopic refractive outcomes in patients with combined cataract and DMEK surgery (triple procedure), there was a higher risk of hyperopic shift in FED eyes with positive pre-operative posterior *Q* values [12]. In our cohort, we did not observe a similar correlation despite negative posterior *Q* values after resolution of the stromal edema. Nevertheless, we assume that resolution of stromal edema is an important contributing factor to the hyperopic shift observed in triple DMEK due to its impact on IOL power calculation. Therefore, performing cataract surgery in FED patients prior to the development of corneal decompensation might limit the factor of inaccurate IOL power calculation.

Factors such as performance of cataract surgery at different institutions, heterogeneous time intervals between cataract surgery and DMEK surgery as well as differences concerning duration and extent of corneal decompensation represent limitations of our study. Furthermore, it has to be validated whether our findings obtained from pseudophakic patients with FED can be applied to other causes of endothelial dysfunction, like pseudophakic bullous keratopathy.

Despite a slight tendency toward a postoperative hyperopic shift, we could demonstrate that changes in SE were statistically not significant. Our analysis of corneal parameters by Scheimpflug tomography points out the relevance of changes in posterior corneal astigmatism for refractive outcome. However, an unexpected refractive outcome frequently reported in patients undergoing triple DMEK [1, 12] seems to be less common after DMEK in pseudophakic patients.
